# Allergic diseases of the skin and drug allergies – 2006. Cord blood 25-hydroxyvitamin D3 and allergic disease during infancy

**DOI:** 10.1186/1939-4551-6-S1-P96

**Published:** 2013-04-23

**Authors:** Anderson Phillip Jones, Debra Palmer, Guicheng Zhang, Susan Prescott

**Affiliations:** 1 School of Paediatrics and Child Health, University of Western Australia, Perth, Australia; 2 Immunology, Princess Margaret Hospital, Australia

## Background

There has been growing interest in vitamin D insufficiency as a predisposing factor for allergy development based on immunoregulatory properties and epidemiological studies. The aim of this study was to investigate the association between vitamin D exposure *in utero* and allergic outcomes in the first year of life.

## Methods

Cord blood (CB) vitamin D was measured in 231 high risk infants from an Australian prospective birth cohort. CB 25(OH)D3 concentration was analysed in relation to maternal vitamin D intake and the development of infant eczema, allergen sensitization and IgE-mediated food allergy.

## Results

Maternal intake of supplemental vitamin D was significantly correlated with CB 25(OH)D3 concentration (rho = 0.244, *p* = 0.003) while dietary vitamin D did not influence CB levels. There was significant seasonal variation in CB 25(OH)D3 concentration suggesting that sunlight exposure was an important determinant. Lower CB vitamin D status was observed in infants that developed eczema (*p* = 0.018), and eczema was significantly more likely in those with concentrations < 50 nmol/L compared with >75 nmol/L (OR 2.66; 95% CI 1.24 – 5.72; *p* = 0.012). This association remained significant after adjustment for multiple confounding factors. The associations between CB 25(OH)D3 concentration and allergen sensitization, IgE-mediated food allergy and eczema severity (SCORAD) were not significant.

## Conclusions

Reduced vitamin D status in pregnancy may be a risk factor for the development of eczema in the first year of life, reinforcing the need to explore the role of vitamin D exposure during development for disease prevention.

